# PATZ1 expression correlates positively with BAX and negatively with BCL6 and survival in human diffuse large B cell lymphomas

**DOI:** 10.18632/oncotarget.10993

**Published:** 2016-08-01

**Authors:** Renato Franco, Giosuè Scognamiglio, Elena Valentino, Michela Vitiello, Antonio Luciano, Giuseppe Palma, Claudio Arra, Elvira La Mantia, Luigi Panico, Valentina Tenneriello, Antonello Pinto, Ferdinando Frigeri, Gaetana Capobianco, Gerardo Botti, Laura Cerchia, Annarosaria De Chiara, Monica Fedele

**Affiliations:** ^1^ Surgical Pathology Unit, National Cancer Institute ‘Fondazione Giovanni Pascale’, IRCCS, Naples, Italy; ^2^ Pathology Unit, Second University of Naples, Naples, Italy; ^3^ Institute of Experimental Endocrinology and Oncology (IEOS), National Research Council (CNR), Naples, Italy; ^4^ Animal Facility, National Cancer Institute ‘Fondazione Giovanni Pascale’, IRCCS, Naples, Italy; ^5^ Pathology Unit, Hospital ‘S.G. Moscati’, Avellino, Italy; ^6^ Haematology-Oncology and Stem Cell Transplantation Unit, Department of Hematology, National Cancer Institute, Fondazione ‘G. Pascale’, IRCCS, Naples, Italy

**Keywords:** non-Hodgkin lymphomas, prognostic marker, PATZ1, tissue-microarray, DLBCL

## Abstract

Non-Hodgkin lymphomas (NHLs) include a heterogeneous group of diseases, which differ in both cellular origin and clinical behavior. Among the aggressive malignancies of this group, the diffuse large B-cell lymphomas (DLBCLs) are the most frequently observed. They are themselves clinically and molecularly heterogeneous and have been further sub-divided in three sub-types according to different cell of origin, mechanisms of oncogenesis and clinical outcome. Among them, the germinal center B-cell-like (GCB) derives from the germinal center and expresses the BCL6 oncogene. We have previously shown that Patz1-knockout mice develop B-cell neoplasias, suggesting a tumor suppressor role for PATZ1 in human NHLs. Here, by immunohistochemical analysis of a tissue-microarray including 170 NHLs, we found that PATZ1 nuclear expression is down-regulated in follicular lymphomas and DLBCLs. Moreover, consistent with our previous results showing a PATZ1-dependent regulation of BCL6 and BAX transcription, we show that low PATZ1 nuclear expression significantly correlates with high BCL6 expression, mainly in DLBCLs, and with low BAX expression, also considering separately follicular lymphomas and DLBCLs. Finally, by analyzing overall and progression-free survival in DLBCL patients that underwent rituximab plus cyclophosphamide, doxorubicin, vincristine, and prednisone chemotherapy, low levels of PATZ1 were significantly associated to a worst outcome and demonstrated an independent prognostic factor in multivariate analysis, including known prognostic factors of DLBCL, IPI score and cell of origin (GCB/non-GCB). Therefore, we propose PATZ1 as a new prognostic marker of DLBCLs, which may act as a tumor suppressor by enhancing apoptosis through inhibiting and enhancing transcription of BCL6 and BAX, respectively.

## INTRODUCTION

Non-Hodgkin Lymphoma (NHL), including 85% of B-Cell Lymphomas represents the most common haematological malignancy disorder [1]. NHLs are a heterogeneous group of diseases, including histotypes related to their origin within the lymphoid system and its different cellular components [1]. In addition, histotypes relate to different clinical behaviour, being indolently growing tumours, e.g. Follicular Lymphoma (FL) or aggressive malignancies, e.g. Diffuse Large B-Cell Lymphoma (DLBCL) [2]. DBCLs and FLs accounted for more than 60% of NHLs, but many other less- common and often rare sub-types of NHL, requiring complex diagnostic approaches and management, have been introduced in the recent 2008 WHO classification [3, 4]. Although NHLs have been recently classified into different groups according to clinicopathological homologies, each of these entities is also heterogeneous with regards to clinical presentation and outcome, often making clinical management of these patients difficult [2]. Recent biotechnological advances concurred to better define histotypes subsets correlated with diverse outcome [5, 6]. Particularly in the case of DLBCL, three subgroups according to cellular origin have been demonstrated, with different clinical outcome; Germinal Centre B-cell like (GCB), Activated B-Cell like (ABC) and type 3 [5, 6]. Regarding FLs, they can be classified into grades according to the morphological identification of neoplastic centroblasts, relating to possible transformation into more aggressive DLBCL [7]. Albeit potentially powerful as prognostic biomarkers, genetic markers and gene expression profiles cannot readily be introduced into clinical routine practice due to technical issues [8]. Thus, for the sub-classification of DLBCLs an immunohistochemical approach has been proposed, although correspondence to gene profiling data is imprecise, being applicable to discriminate GCB cases from non-GCB cases, including ABC and type 3 subgroups [9, 10]. However, gene expression profiling has also revealed that DLBCL sub-groups have distinct oncogenic mechanisms that respond differently to therapies [11]. Two oncogenic mechanisms that appear to be active in GCB DLBCLs are the prevention of apoptosis and the blocking of terminal differentiation [12]. One of the master oncogenes in this DLBCL sub-group is B cell lymphoma 6 (BCL6), which is involved in several cell processes that can impact on the ability of the B-cell to differentiate and proliferate [12]. Recently, we have shown that the POZ/domain and kruppel zinc finger (POK) transcription factor PATZ1 plays a crucial role in the negative autoregulation of BCL6 expression, and that Patz1-knockout mice develop a thymus neoplastic phenotype caused by BCL6 overexpression, suggesting that PATZ1 could be involved in BCL6 expressing human B cell lymphomas [13]. *PATZ1* gene has been associated to different types of cancer sometimes working as a tumor suppressor and sometimes as an oncogene, depending on the cellular context [14–17]. As for other members of the POK family, PATZ1 plays key roles in development and cancer through its involvement in a variety of cellular processes, including cell proliferation, DNA repair, senescence, apoptosis and differentiation [16–22]. In most of these processes it acts upstream and downstream of p53, suggesting it could be a key regulator of the p53 pathway [16, 18, 22]. The *TP53* tumor suppressor gene is directly regulated by BCL6, which represses its transcription in GCB DLBCLs [23] and its inactivation has been correlated with a poor prognosis in patients with DLBCL treated with cyclophosphamide, hydroxydaunorubicin, vincristine, and prednisone (CHOP) therapy or rituximab plus CHOP (R-CHOP) [24].

Here, we performed immunohistochemical staining of a Tissue-Micro-Array (TMA), including 170 cases of NHLs, to investigate the expression of PATZ1, BCL6 and the p53/PATZ1 target BAX and their reciprocal correlations with respect to the different histotype and sub-type.

To finally assess the impact of PATZ1 expression on patients' outcome, we analysed overall (OS) and progression-free (PFS) survival of DLBCL patients treated with R-CHOP, including a public dataset of 470 patients from the MD Anderson Cancer Center [25], other than 65 patients from our TMA series. We found that low PATZ1 (either gene or protein) expression significantly correlates with poor survival, suggesting PATZ1 as a negative prognostic marker for this heterogeneous malignancy.

## RESULTS

### Tissue-micro-array design and Clinicopathological data

One hundred and seventy tissue samples were used for a TMA building, using three tissue cores taken from two to three discrete but representative regions of each single case. The main clinicopathological data of the 170 cases of NHL included in the TMA are set out in Table 1. The series included 83 (48.8%) males and 87 (51.2%) females, with a mean age of 59.9 +/− 14 (Standard deviation). In our histological samples, there were 70 (41.2%) FLs and 100 (58.8%) DLBCLs. Moreover, DLBCLs included 47 GCB (47%) and 53 non-GCB (53%).

**Table 1:** Clinicopathological characteristics

Biomarker		Tot (*n* = 170)
Gender	F	87 (51.2%)
	M	83 (48.8%)
Median Age	≤ 60	80 (47.1%)
	> 60	90 (52.9%)
Istotype	FL	70 (41.2%)
	DLBCL	100 (58.8%)
DLBCL sub-type	non-GCB	53 (53%)
	GBC	47 (47%)

### PATZ1 is absent or mislocalized in NHLs

Our previous studies in mice have suggested that PATZ1 may act as a tumor suppressor in lymphomagenesis, particularly in the development of NHLs [13]. To explore this potential role in human patients, we analyzed expression of PATZ1 by immunohistochemistry in the TMA described above.

**Figure 1:**
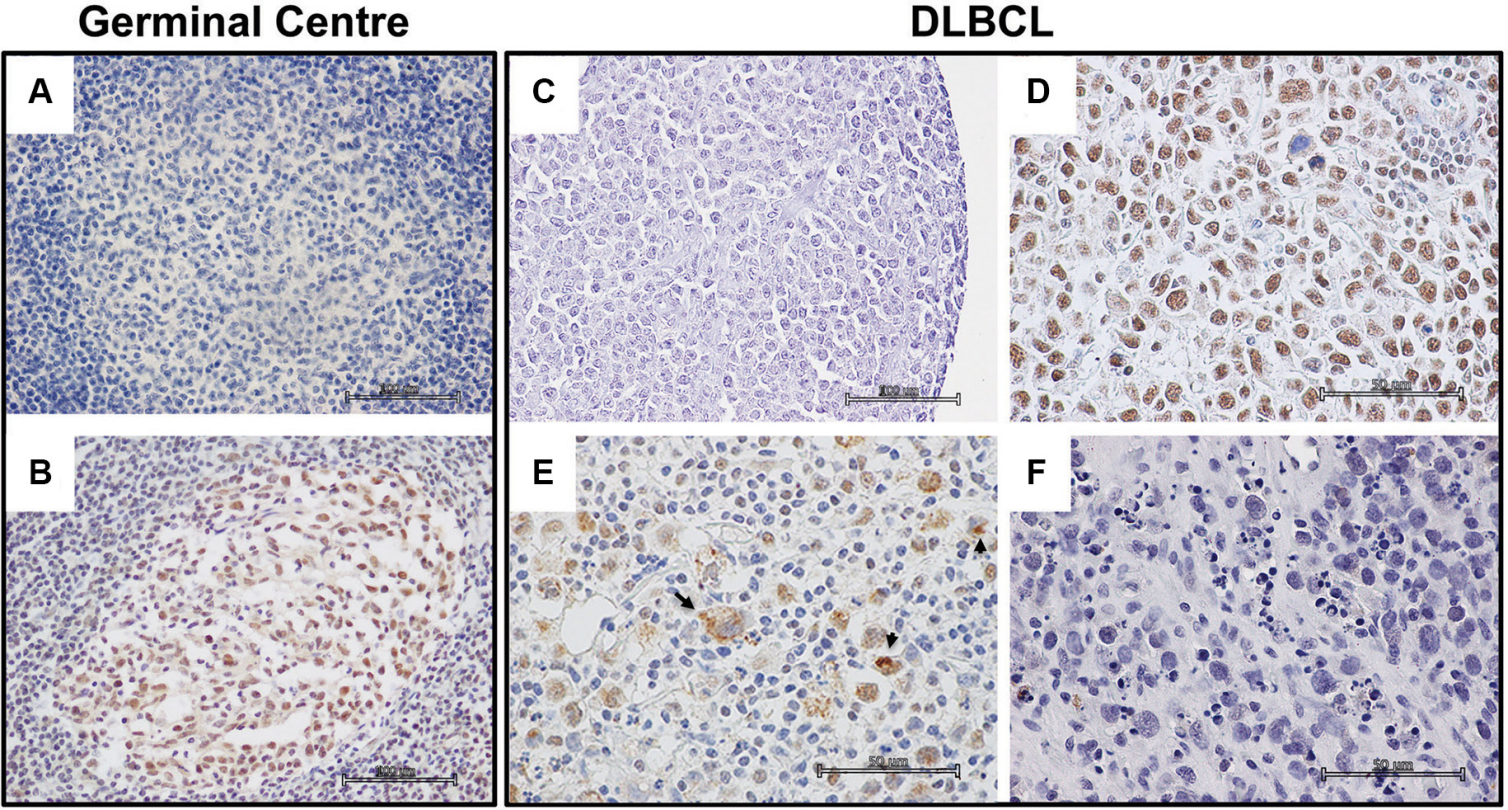
PATZ1 immunohistochemical expression in human Germinal Centre and DLBCLs (**A**–**B**) Representative images of a germinal centre in a tonsil follicular hyperplasia control: (A) No antibody negative control; (B) PATZ1 staining. (**C**–**F**) Representative images of different PATZ1 expression and sub-cellular localization in DLBCLs: (C) No antibody negative control; (D) PATZ1 nuclear expression; (E) PATZ1 nuclear/cytoplasmic expression, arrows indicate cells with cytoplasmic staining; (F) PATZ1 negative expression. Scale bars: 100 μm in A, B and C; 50 μm in D, E and F.

In the tonsil follicular hyperplasia control (FH), PATZ1 was expressed exclusively in the nucleus of the GC cells (Figure 1A–1B, Table 2). Conversely, in both FLs and DLBCLs PATZ1 expression was heterogeneous, being nuclear, cytoplasmic, both cytoplasmic and nuclear or completely absent (Figure 1C–1F). In particular, the frequency of cells with nuclear expression of PATZ1 was low in most FLs (59%) and DLBCL-GCB (53%) (Table 2), supporting the possible tumor suppressor role for PATZ1 mainly in the FL and DLBCL-GCB sub-types. The appearance of a PATZ1 cytoplasmic staining in neoplastic samples, which is suggestive of a loss of function of the protein, is consistent with previous reports showing similar results in testicular and thyroid tumors [17, 26]. Therefore, hereafter we only refer to the PATZ1 nuclear staining.

**Table 2:** PATZ1 nuclear expression and sub-cellular localization

Hystotype	Nuclear anti-PATZ1 reactivity			PATZ1 sub-cellular localization			Negative^a^
	N.	(−)	(+)	Nuclear	Nucl/Cyt	Cytosol	
**FH**	4	0	4 (100%)	4 (100%)	0	0	0
**FL**	70	41 (59%)	29 (41%)	13 (19%)	16 (23%)	5 (7%)	36 (51%)
**DLBCL**	100	45 (45%)	55 (55%)	29 (29%)	26 (26%)	6 (6%)	39 (39%)
** -non GCB**	53	20 (38%)	33 (62%)	20 (38%)	13 (24.5%)	4 (7,5%)	16 (30%)
** -GCB**	47	25 (53%)	22 (47%)	9 (19%)	13 (28%)	2 (4%)	23 (49%)

a PATZ1 expression absent in both nucleus and cytoplasm.

### Correlation between PATZ1 and either BCL6 or BAX in NHLs

We have previously shown that PATZ1 is a direct transcriptional regulator of BCL6 [13] and BAX [16]. Both BCL6 and BAX play key roles in lymphomagenesis, acting as oncoprotein or tumor suppressor, respectively [27, 28]. In our cohort of samples BCL6 was highly expressed in 64 out of 70 FLs (91.4%) and in 76 out of 100 DLBCL (76%), including 31/53 (58.5%) non-GCB and 45/47 (96%) GCB. Conversely, BAX expression was observed overexpressed in 33 out of 70 FLs (47.1%) and 52 out of 100 DLBCLs (52%), including 33/53 (62%) ABC and 23/47 (49%) GCB (Table 3).

**Table 3:** Correlation between BCL6, BAX and PATZ1 nuclear expression and clinicopathological characteristics

		BCL6		BAX		PATZ1	
		(−)	(+)	(−)	(+)	(−)	(+)
**Age**	< 60	10 (12.5%)	70 (87.5%)	41 (51.3%)	39 (48.7%)	43 (53.8%)	37 (46.2%)
	> 60	20 (22.2%)	70 (77.8%)	44 (48.9%)	46 (51.1%)	43 (47.8%)	47 (52.2%)
**Gender**	M	19 (21.8%)	68 (78.2%)	40 (46%)	47 (54%)	39 (44.8%)	48 (55.2%)
	F	11 (13.3%)	72 (86.7%)	45 (54.2%)	38 (45.8%)	47 (56.6%)	36 (43.4%)
**Histotype**	FL	6 (8.6%)	64 (91.4%)	37 (82.9%)	33 (47.1%)	41 (58.6%)	29 (41.4%)
	DLBCL	24 (24%)	76 (76%)	48 (48%)	52 (52%)	45 (45%)	55 (55%)
**DLBCL Subtype**	non GCB	22 (41.5%)	31 (58.5%)	24 (45.3%)	29 (54.7%)	20 (37.7%)	33 (62.3%)
	GCB	2 (4.3%)	45 (95.7%)	24 (51.1%)	23 (48.9%)	25 (53.2%)	22 (46.8%)

**Figure 2:**
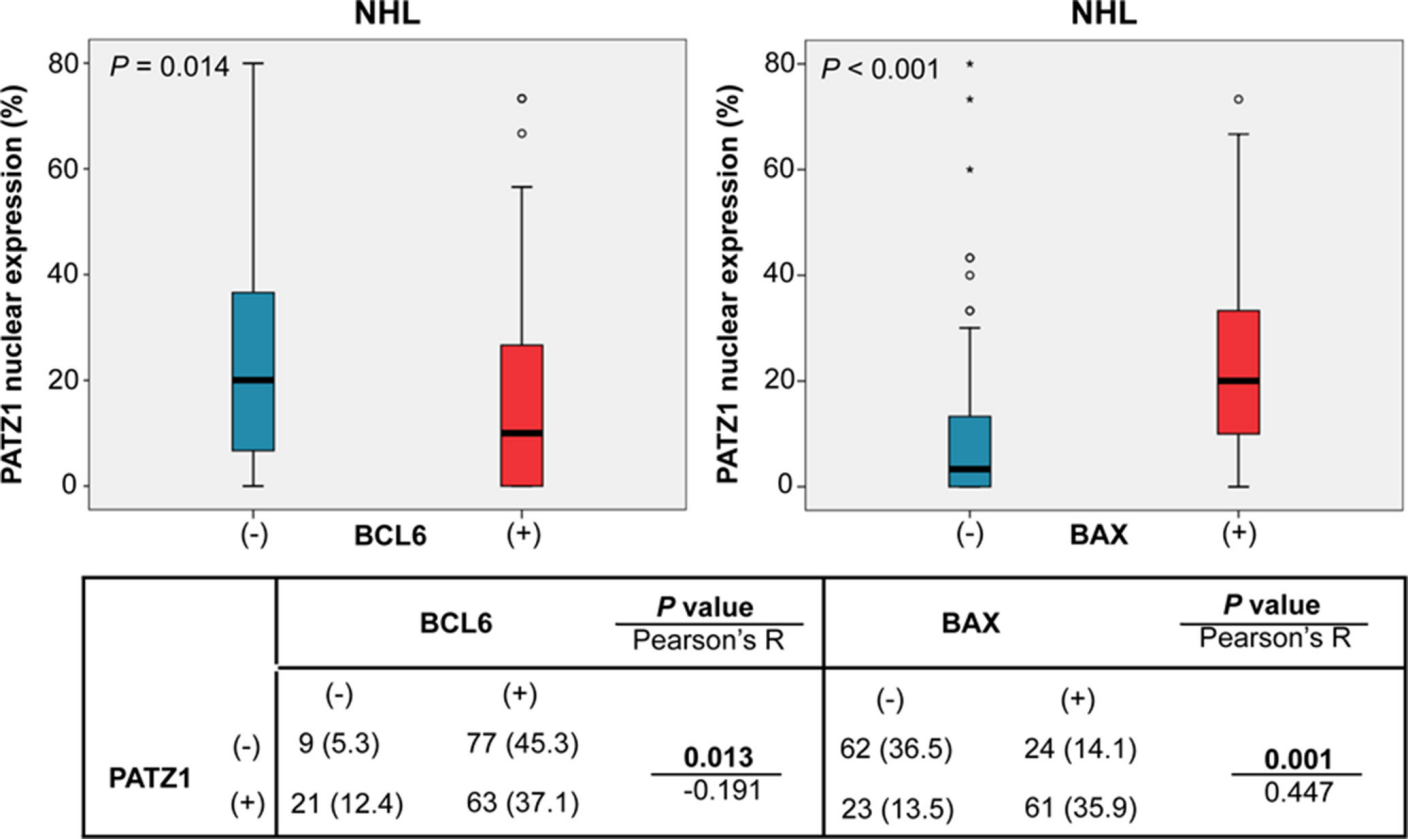
PATZ1, BCL6 and BAX correlation in NHL cohort Boxplot showing correlation between PATZ1 percentage expression and BCL6 (left) and BAX (right) high (+) or absent/low (−) expression. Data were analyzed by Mann-Whitney test and the resulting *p*-value is reported on the left-up corner of each boxplot panel. The table below shows total number (and percentage values in brackets) of samples double positive or single positive for each of BCL6 and PATZ1 (left) or BAX and PATZ1 (right) protein expression. Data were analyzed by the Pearson's *χ*^2^ test and the resulted p-value and R coefficient are reported where indicated.

**Figure 3:**
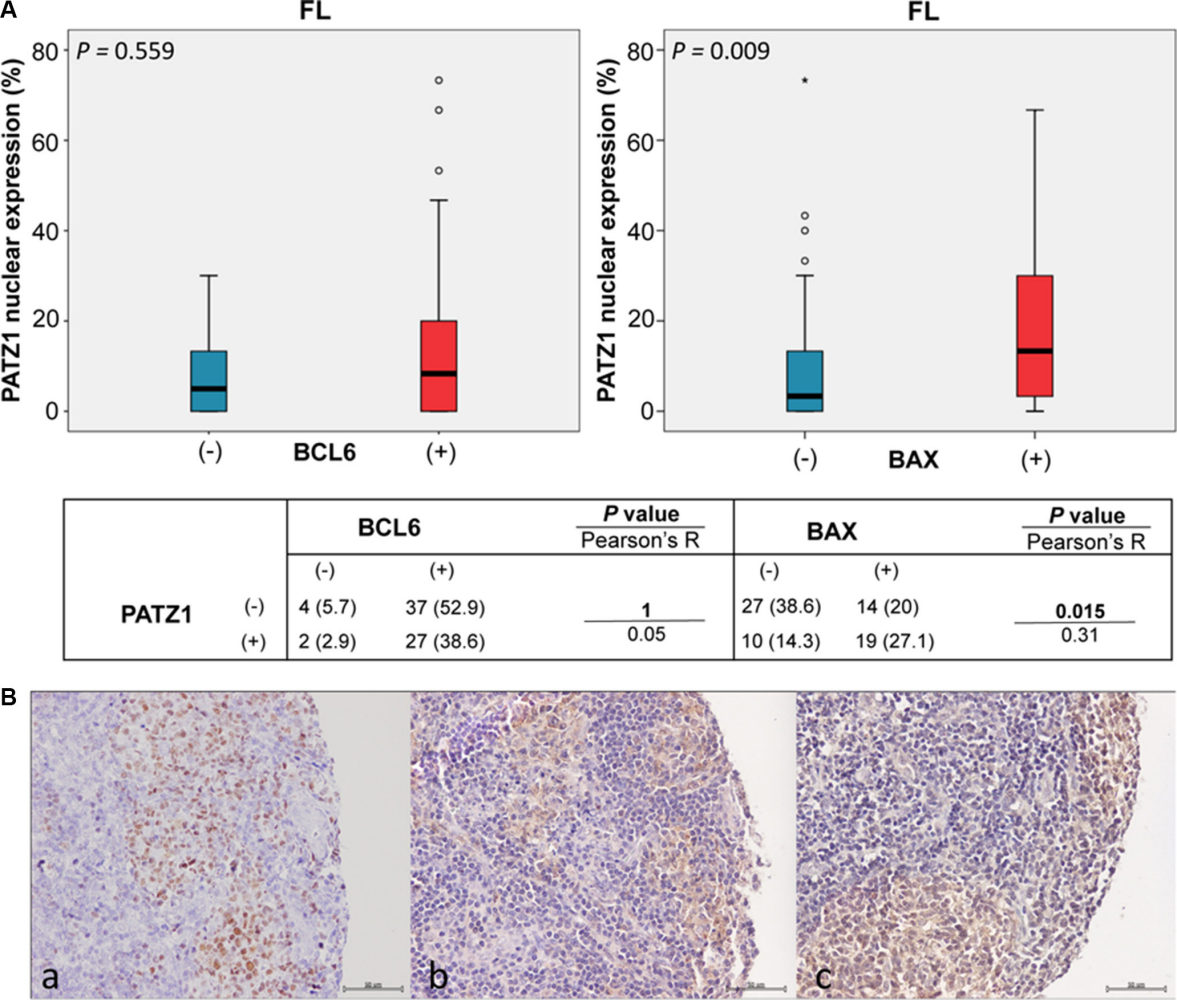
PATZ1, BCL6 and BAX expression and correlations in FLs (**A**) Boxplot of FL cohort: correlation between PATZ1 nuclear expression and BCL6 (left) and BAX (right) high (+) or absent/low (−) expression. Data were analyzed by Mann-Whitney test and the resulting *p*-value is reported on the left-up corner of each boxplot panel. The table below shows total number (and percentage values in brackets) of samples double positive or single positive for each of BCL6 and PATZ1 (left) or BAX and PATZ1 (right) protein expression. Data were analyzed by the Pearson's *χ*^2^ test and the resulted *p*-value and R coefficient are reported where indicated. (**B**) Immunophenotype of a representative case of FL. Serial sections were stained for BCL6 (a), BAX (b) and PATZ1 (c). Positive expression was detected for all three proteins. Scale bars: 50 μm.

**Figure 4:**
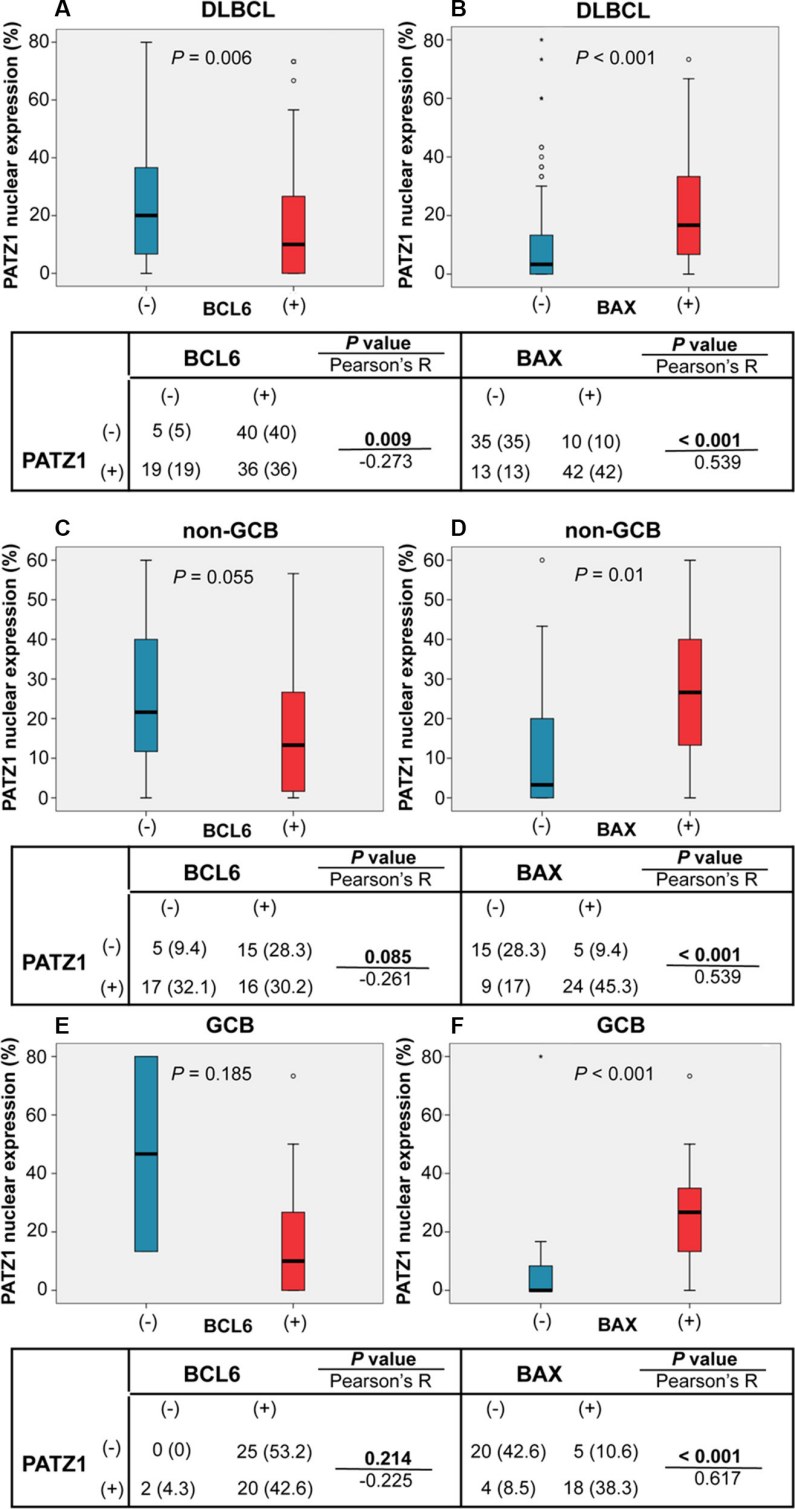
PATZ1, BCL6 and BAX correlations in DLBCLs Boxplot of DLBCL (**A**, **B**) and sub-types non-GCB (**C**, **D**) and GCB (**E**, **F**) cohorts: correlation between PATZ1 nuclear expression and BCL6 (A, C, E) and BAX (B, D, E) high (+) or absent/low (−) expression. Data were analyzed by Mann-Whitney test and the resulting *p*-value is reported in each boxplot panel. The tables below each boxplot show total number (and percentage values in brackets) of samples double positive or single positive for each of BCL6 and PATZ1 (left) or BAX and PATZ1 (right) protein expression. Data were analyzed by the Pearson's *χ*^2^ test and the resulted *p*-value and R coefficient are reported where indicated.

A basic picture of the correlation between the frequency of BCL6, BAX and PATZ1 positive cells and clinicopathological features are shown in Table 3. Interrelation among the frequencies of BCL6, BAX and PATZ1 positive cells was analyzed by both Pearson's exact test on the positive/negative categories reported in Table 4, and Mann-Whitney test on the percentage of positive cells. The results, represented in Figures 2, 3 and 4, showed significant associations between PATZ1 expression and either BCL6 or BAX in different NHL histotypes and DLBCL sub-types:

**Table 4:** Frequencies of the BCL6, PATZ1 and BAX biomarkers in NHLs

Biomarker		Cutoff^a^	Tot
BCL6	(−)	≤ 30	30 (17.6%)
	(+)	> 31	140 (82.4%)
PATZ1 Nuclear	(−)	≤ 11.6	86 (50.6%)
	(+)	> 11.7	84 (49.4%)
PATZ1 Cytoplasmic	(−)	≤ 2	117 (68.8%)
	(+)	> 3	53 (31.2%)
BAX	(−)	≤ 5.0	85 (50%)
	(+)	> 5.1	85 (50%)

a The median immunopositivity has been used as cutoff.

**Figure 5:**
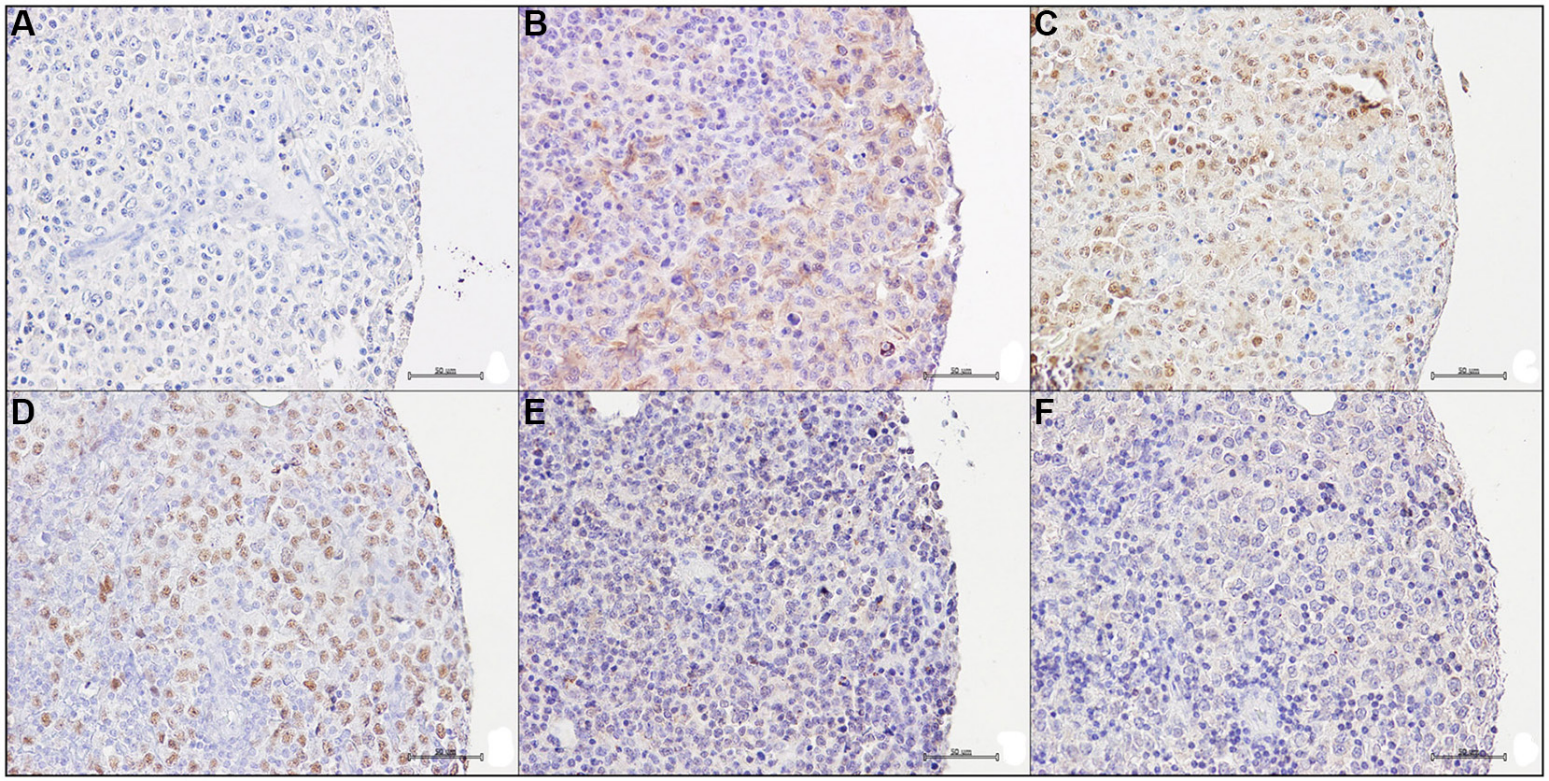
Immunophenotype of DLBCLs Serial sections of two representative cases of DLBCL were stained for BCL6, BAX and PATZ1: Case 1 (**A**, **B**, **C**) is BCL6 negative (A), BAX positive (B) and PATZ1 positive (C); Case 2 (**D**, **E**, **F**) is BCL6 positive (D), BAX negative (E) and PATZ1 negative (F). Scale bars: 50 μm.

*PATZ1 and BCL6* - In the NHL group, BCL6 expression negatively correlates with PATZ1 expression, being PATZ1 more expressed in the BCL6 low samples than in the BCL6 high ones (*p* = 0.013 and 0.014; Pearson and Mann-Whitney tests, respectively) (Figure 2); this correlation is not significant in the FL group (*p* = 1 and 0.559; Pearson and Mann-Whitney tests, respectively) (Figure 3), but it is in the DLBCL group (*p* = 0.009 and 0.006; Pearson and Mann-Whitney tests, respectively), with a trend to be negatively correlated in both non-GCB and GCB sub-types (Figures 4 and 5). These data are consistent with the inhibition of BCL6 transcription by PATZ1 in human DLBCLs.

**Figure 6:**
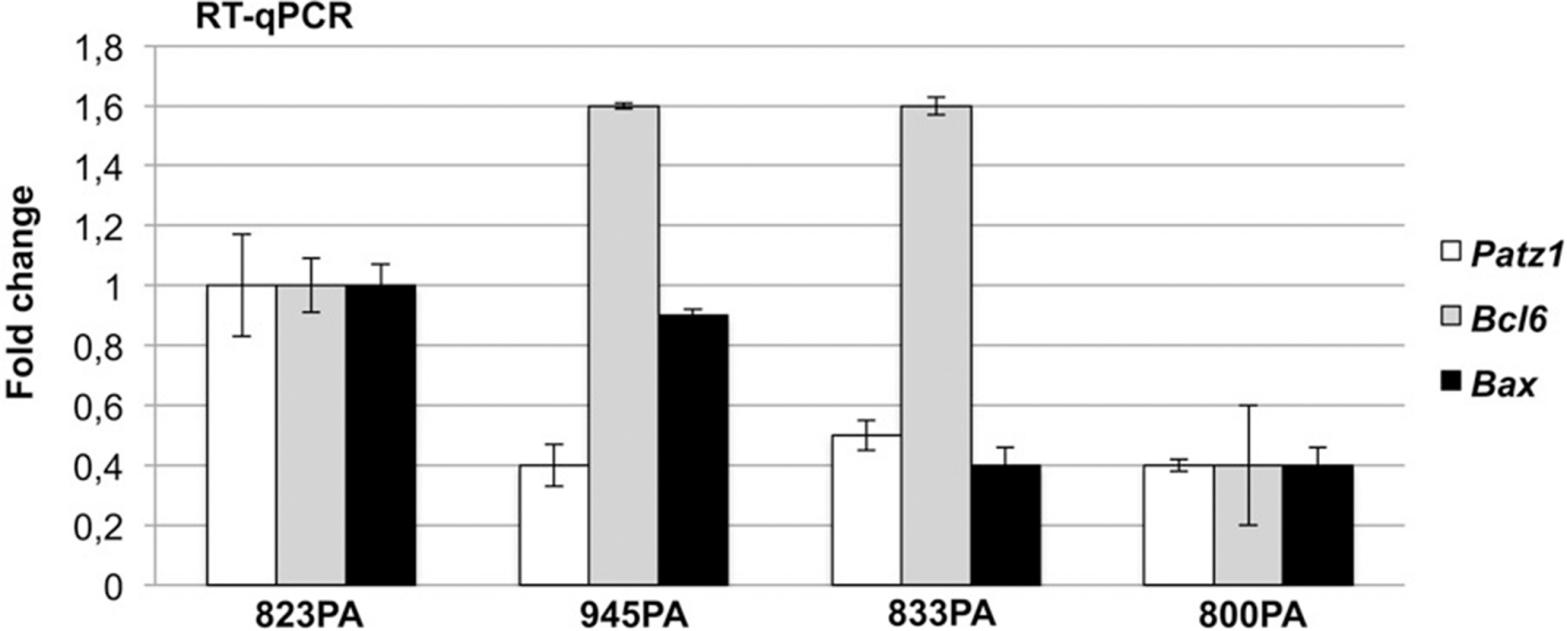
*Bcl6* and *Bax* expression in Patz1-knockout mice RT-qPCR showing expression of *Patz1*, *Bcl6* and *Bax* genes in one healthy spleen (945PA) and two lymphomas (833PA, 800PA) from Patz1+/− mice in comparison with a normal spleen sample from a wild-type syngenic mouse (823PA). Mean values ± SE of a representative experiment performed in duplicate are shown.

*PATZ1 and BAX* - In the NHL group, low PATZ1 expression significantly correlates with low BAX expression (*p* < 0.001 by both Pearson and Mann-Whitney tests) (Figure 2); this correlation is significant both in the FL group (*p* = 0.015 and 0.009; Pearson and Mann-Whitney tests, respectively) (Figure 3) and in the DLBCL group (*p* < 0.001 by both Pearson and Mann-Whitney tests), with non-GCB (*p* = 0.001 and 0.01; Pearson and Mann-Whitney tests, respectively) and GCB (*p* < 0.001 by both Pearson and Mann-Whitney tests) (Figures 4 and 5). These data are consistent with the enhancement of BAX transcription by PATZ1 in human NHLs of both FL and DLBCL sub-type, and suggest that downregulation of PATZ1 may cause downregulation of BAX in NHLs. They also suggest that downregulation of BAX could be an additional mechanism of lymphomagenesis, other than BCL6 overexpression, in Patz1-knockout mice. To support such hypothesis we analyzed, by qRT-PCR, *Bax* and *Bcl6* expression in spleen samples of 3 Patz1+/− mice, including one healthy spleen and two DLBCLs, compared to normal spleen from a wild-type mouse. As shown in Figure 6, in all Patz1+/− samples analyzed downregulation of *Bax* was observed. In particular, in one lymphoma (833PA) it was accompanied by overexpression of *Bcl6* and in the other one (800PA) by downregulation of *Bcl6*, indicating a molecular heterogeneity in Patz1-dependent lymphomagenesis, sometime involving BCL6 and more often BAX. Consistent with previous data [13], also the Patz1+/− healthy spleen (945PA) showed overexpression of *Bcl6,* but it expressed *Bax* at the same levels of the wild-type sample, suggesting that the PATZ1-dependent enhancement of the *Bax* gene expression in this cell context occurs only in malignant cells likely as a way to lead malignant cells towards apoptosis.

### PATZ1 downregulation correlates with poor survival in DLBCL patients treated with R-CHOP

**Figure 7:**
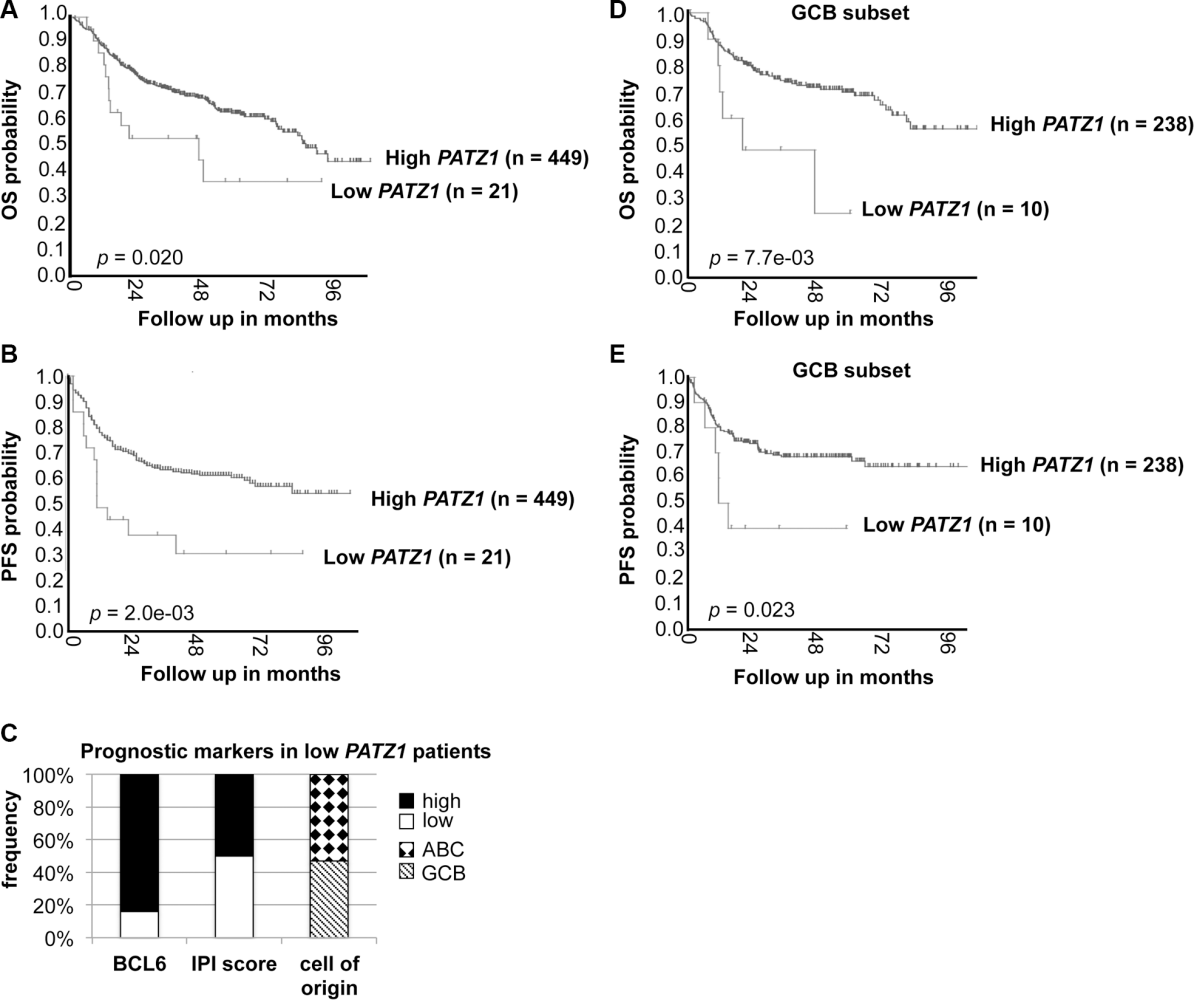
*PATZ1* gene expression correlates with survival in DLBCL patients treated with R-CHOP (**A**) Overall (OS) and (**B**) Progression-Free (PFS) Kaplan–Meier survival curves of DLBCL patients treated with R-CHOP, adapted from the genomic Analysis and Visualization platform (http://r2.amc.nl). Note that patients with lower *PATZ1* expression have a worse OS and PFS survival rate than patients with higher *PATZ1* expression as assessed by LogRank test (*p* < 0.05 and 0.01, respectively). (**C**) BCL6 protein expression, IPI score and cell of origin in patients with low *PATZ1* expression in the PFS survival curve. Cutoff score of 33% was used for BCL6, as previously reported (25). (**D**) OS and (**E**) PFS Kaplan-Meier survival curves of the GCB subset of DLBCL patients shown in A and B. Note that patients with lower *PATZ1* expression still have a worse OS and PFS survival rate compared to patients with higher *PATZ1* expression, as assessed by Log Rank test (*p* < 0.01 and 0.05, respectively).

By analyzing a public dataset through microarray analysis and visualization platform (http://r2.amc.nl), we found that *PATZ1* low expression correlates with poor survival in a cohort of 470 human DLBCL patients treated with R-CHOP immunochemotherapy [25]. In fact, as shown in Figure 7A–7B, both OS and PFS Survival curves indicate a statistically significant worst outcome in patients with lower levels of *PATZ1* in the neoplastic samples. Indeed, DLBCL patients with high *PATZ1* levels had significantly better OS and PFS compared with DLBCL patients with low *PATZ1* (*p* = 0.020 and 0.002, respectively; Figure 7A–7B). The median OS for 449 patients with high *PATZ1* expression was 83 months, in contrast to 45 months for 21 patients with low *PATZ1*. The median PFS for patients with high *PATZ1* DLBCL was > 96 months versus 11 months for patients with low *PATZ1* DLBCL. The 5-year PFS was 61% for DLBCL patients with high *PATZ1* versus 29% with low *PATZ1*. These data suggest that PATZ1 may be a predictive marker that stratifies DLBCL patients treated with R-CHOP. By analyzing, through the one way analysis of the variance (ANOVA), the clinical and protein expression data available for these patients, we could not find any significant correlation between PATZ1 expression and other well recognized prognostic factors, such as International Prognostic Index (IPI) code and BCL6 protein expression, except for a positive correlation with the GCB cell of origin (*p* = 0.05), which has been indicated as a favorable prognostic factor for DLBCLs treated with R-CHOP [25]. However, by restricting our analysis to the patients in which low expression of PATZ1 was correlated with a worse PFS following R-CHOP therapy (Figure 7B and Table Supplementary Digital Content 1, showing clinicopathological features and follow-up in low *PATZ1* patients), we found that most patients (84%) have high frequency (above the established cutoff of 33) of BCL6 positive cells, supporting in these patients the negative regulation of BCL6 expression by PATZ1, but no evident correlation was observed with IPI code and cell of origin (Figure 7C), as well as with protein expression of FOXP1 and MUM1, higher values of which have been previously associated with inferior PFS in DLBCL patients treated with R-CHOP [25]. Conversely, a trend for a positive correlation with CD10 and GCET1 protein expression, higher values of which have been shown positive effect on PFS [25], was observed (Table Supplementary Digital Content 1). Interestingly, PATZ1 low expression is a negative prognostic marker also if we only consider GCB DLBCL tumors (Figure 7D, 7E), meaning that GCB DLBCL tumors exhibiting low levels of PATZ1 can be associated with worse prognosis. In addition, in a multivariate analysis, including known prognostic factors IPI and cell of origin (GCB/non-GCB), low expression of PATZ1 resulted an independent prognostic factor for PFS (HR = 0.40, 95% CI = 0.22–0.72, *P* = 0.002) and OS survival (HR = 0.42, 95% CI = 0.23–0.76, *P* = 0.00) (Table 5).

**Table 5:** Multivariate analysis comparing PATZ1, cell of origin and IPI in the dataset shown in Figure 7

Variables	Overall Survival		Progression-Free Survival	
	HR (95% CI)	*P* values	HR (95% CI)	*P* values
PATZ1	0.42 (0.23–0.76)	0.00	0.40 (0.22–0.72)	0.002
Cell of origin	0.62 (0.45–0.85)	0.00	0.56 (0.41–0.78)	0.001
IPI	3.65 (2.53–5.29)	0.00	2.71 (1.85–3.99)	0.000

**Figure 8:**
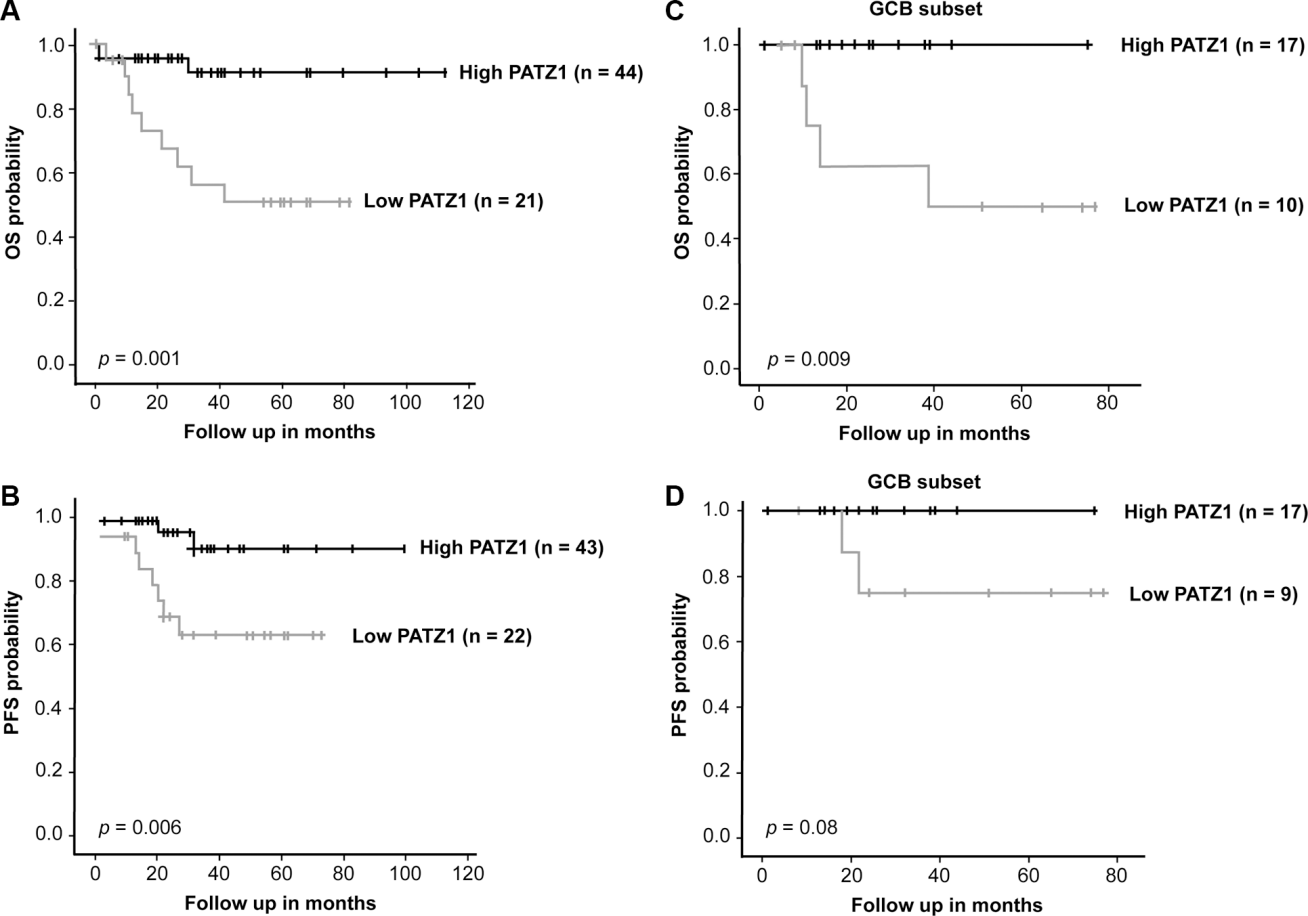
PATZ1 protein expression correlates with survival in DLBCL patients treated with R-CHOP (**A**) Overall (OS) and (**B**) Progression-Free (PFS) Kaplan-Meier survival curves of R-CHOP-treated DLBCL patients from our TMA series. Note that patients with lower PATZ1 protein expression have a significant worse OS and PFS survival rate than patients with higher PATZ1 expression, as assessed by LogRank test (*p* < 0.001 and 0.01, respectively). (**C**) OS and (**D**) PFS Kaplan-Meier survival curves of the GCB subset of DLBCL patients shown in A and B. Note that patients with lower PATZ1 protein expression have a worse OS and a trend to a worse PFS survival rate compared to patients with higher PATZ1 expression (Log Rank test, *p* < 0.01 and = 0.08, respectively).

To strengthen our findings, we also correlated PATZ1 protein expression to OS and PFS in our TMA series, considering 65 patients submitted to R-CHOP treatment. As reported in Figure 8, Kaplan-Meier survival analysis shows that patients with low PATZ1 protein expression had a significant shorter OS and PFS than high PATZ1 protein expression patients' group (*p* = 0.001 and 0.006, respectively). Also in this case, if we restrict the analyses to GCB DLBCL tumors, low PATZ1 protein expression is still associated to a worse outcome (Figure 8C, 8D). Finally, also in this series, PATZ1 low expression was demonstrated as an independent prognostic factor in multivariate analysis in terms of PFS (HR = 0.08, 95% CI = 0.02–0.42, *P* = 0.00) and OS (HR = 0.14, 95% CI = 0.04–0.53, *P* = 0.00) (Table 6).

**Table 6:** Multivariate analysis comparing PATZ1, cell of origin and IPI in the dataset shown in Figure 8

Variables	Overall Survival		Progression-Free Survival	
	HR (95% CI)	*P* values	HR (95% CI)	*P* values
PATZ1	0.14 (0.04–0.53)	0.00	0.08 (0.02–0.42)	0.00
Cell of origin	0.72 (0.22–2.36)	0.59	0.50 (0.12–2.06)	0.34
IPI	3.58 (1.12–11.47	0.03	6.27 (1.58–24.90)	0.01

All together these data confirm PATZ1 low expression as a negative prognostic marker in DLBCL patients treated with R-CHOP.

## DISCUSSION

An important role in the pathogenesis of some aggressive NHLs, such as DLBCLs, appears to be played by the deregulation of the BCL6 gene. Various evidences indicate that BCL6 plays an important role in the development of all NHL derived from germinal center. Indeed, it is necessary for the development of GC and, in physiological conditions, prevents apoptosis in order to promote the rearrangement of the immunoglobulin genes for the formation of antibodies. Similarly, in NHLs the BCL6 anti-apoptotic role could play a pivotal role in the malignancy development. BCL6 constitutive expression in DLBCLs is mainly the consequence of BCL6 gene rearrangements and activating mutations that target the 5′ regulatory region of this gene [27, 29–31]. However, there is evidence showing that BCL6 expression in lymphoma is often independent from the corresponding chromosomal alterations, suggesting that mechanisms other than gene rearrangements or mutations can deregulate BCL6 expression in DLBCLs [32, 33]. On this basis, a possible role in the pathogenesis of DLBCL could be attributed to the downregulation of the transcription factor PATZ1. Indeed, in knockout mice for the *Patz1* gene, BCL6 is overexpressed and, through *in vitro* experiments in human B cells, a direct role for PATZ1 in the negative regulation of BCL6 expression has been demonstrated, suggesting that the up-regulation of BCL6, induced by the reduced expression of Patz1, could cause the neoplastic phenotype in Patz1-knockout mice [13]. BCL6 is involved in the repression of p53 expression [23], suggesting that PATZ1 downregulation could be indirectly involved in downregulating p53 function. However, several lines of evidence indicate a direct role of PATZ1 in the p53 pathway [16–18, 22]. We and others have recently shown that PATZ1 can interact with p53 and interfere with its transcriptional activity [16, 22]. Indeed, we found that it can directly bind and regulate expression of p53 target genes, including BAX, a pro-apoptotic member of the BCL2 family, enhancing apoptosis or cell survival based on the cellular context [16]. Notably, despite a relatively low percentage (∼ 20%) of TP53 somatic mutations in DLBCLs [34], 66% of DLBCLs show reduced levels of p53 targets [35], suggesting that molecular mechanisms other than p53 mutations could be frequently involved in acquiring apoptosis resistance in these malignancies. BAX itself has been found mutated with loss of function in several cell lines of human hematopoietic malignancies, indicating that this pro-apoptotic protein can function as a tumor suppressor in these diseases [28].

In the present work, by TMA immunostaining analyses, using cutoff criteria based on the median percentage of positive cells, we confirmed the existence of a negative correlation between PATZ1 and BCL6 in DLBCL, indicating that the upregulation of BCL6 in this NHL sub-group could be linked to a downregulation of PATZ1. Conversely, a positive correlation between PATZ1 and BAX resulted in both DLBCLs and FLs. Therefore, even if the coefficients of correlation were relatively low, based on the significance obtained by two different statistical tests (Mann-Whiney and Pearson's exact tests), we could assume that in a group of DLBCLs, the upregulation of BCL6 may be due to the downregulation of PATZ1, whichcould also be directly responsible for the downregulation of BAX in the same group of lymphomas, likely resulting in an anti-apoptotic effect. Moreover, as predicted by Kaplan Meier curves for evaluation of OS and PFS in DLBCL patients treated with R-CHOP, using different criteria, based either on RNA or protein expression, low levels of PATZ1 correlate with a worse outcome. Interestingly, most of the patients showing worse outcome and low levels of PATZ1 showed high expression of BCL6. This is quite surprising because BCL6-positivity is considered an indicator of longer PFS and OS in DLBCL patients undergoing R-CHOP therapy [36], but it is consistent with our findings of a negative correlation between PATZ1 and BCL6, which is in agreement with the role of PATZ1 in the negative regulation of its expression. Chromosomal translocations involving BCL6 are present in at least one third of DLBCLs and BCL6 activating mutations are not rare [37]. Thus, it would be of interest to know the translocation/mutational status of BCL6 in patients according to the High/Low PATZ1 status. In fact, a reduced frequency of translocated/mutated BCL6 in Low PATZ1 cases would support a direct involvement of the reduced PATZ1 in determining higher levels of BCL6.

Prognostic models based on pre-treatment characteristics, such as the IPI and clinicopathologic parameters, including cell of origin, are currently used to predict outcome in DLBCL [38]. In addition, different combinations of specific markers have been suggested as prognostic algorithms for DLBCL patients [25]. We have analyzed the expression of some of these prognostic clinicopathologic parameters and markers in the subset of patients with low levels of PATZ1, which shows unfavorable outcome before 5 years post therapy, finding no differences related to IPI code, cell of origin of the tumor (GCB vs non-GCB), FOXP1 and MUM1 protein expression. Therefore, PATZ1 expression appears to have a prognostic value independent from these known prognostic markers. Conversely, a trend for a positive correlation with CD10 and GCET1 protein expression was observed, suggesting a functional dependence between PATZ1 and these proteins. The prognostic value of PATZ1, independently from cell of origin and IPI, was however assessed by a multivariate analysis on the whole set of samples of each database studied.

In conclusion, we have confirmed a crucial role for PATZ1 in human lymphomas, showing it behaves as a tumor suppressor gene in a subset of NHLs and could be useful as an independent prognostic marker in BCL6-positive samples of DLBCL patients to gain further insight on the possible success of the R-CHOP therapy.

## MATERIALS AND METHODS

### Patients cohorts

We performed a retrospective evaluation of the patients with histologically confirmed NHL. A cohort of 170 cases of NHLs, including 100 DLBCLs and 70 FLs, were collected from the Department of Pathology of National Cancer Institute “Fondazione G. Pascale”, Naples and from Pathology Unit of San Giovanni Moscati Hospital, Avellino. The cases, diagnosed between 2003 and 2011, were included in this study on the basis of the availability of diagnostic paraffin blocks that were thick enough to provide a minimum of 50 sections.

Informed consent for the scientific use of biological material was obtained from all patients and the work has been approved by the local Ethical Committee (CEI 556/10 of 12/3/2010).

All cases were reviewed according to WHO classification criteria, using standard tissue sections and immunohistochemical analysis. In addition, DLBCLs were further sub-classified in GCB and non-GCB DLBCL, including ABC and type-3 cases, through the immunohistochemical panel suggested by Choi et al. [39].

For PATZ1 mRNA expression analysis using the Genomics Analysis and Visualization platform (http://r2.amc.nl) a cohort of 470 DLBCL patients treated with R-CHOP immunochemotherapy was selected as previously reported [25].

### Tissue-micro-array and immunohistochemical study

One hundred and seventy tissue samples were used for a TMA building, using three tissue cores (diameter 0.6 mm) taken from two to three discrete but representative regions of each single case. Tissue cylinders with a diameter of 0.6 mm were punched from morphologically representative tissue areas of each donor tissue block and brought into one recipient paraffin block (3 × 2.5 cm) using a semiautomatic tissue array instrument (Galileo TMA CK-3500, Integrated Systems Engineering, Milan, Italy). Clinicopathological data are listed in Table 1. Immunohistochemical staining was used to classify NHL according to conventional criteria [4]. In addition, immunohistochemical staining was performed on TMA slides (4–5 μm) in order to evaluate expression of PATZ1 (costom clone R1P1, Primm), BCL6 (clone GI191E/A8, Ventana Medical Systems, Tucson, AZ) and BAX (ab 7977, Abcam, Cambridge, UK). The endogenous peroxidase activity was blocked by incubating the sections with about 150 μl of Novocastra Peroxidase Block (RE7101; 3% H_2_O_2_) for 10 min at room temperature, followed by 2 washes (5 min/each) in TBS/Tween buffer. The formation of non-specific binding between the antibodies and endogenous proteins was reduced by incubating the sections with about 150 μl of Novocastra Protein Block (10% FBS) for 10 min at room temperature, followed by 2 washes (5 min/each) in TBS/Tween buffer. After protein block, slides were incubated with about 150 ml of primary antibodies for 1 hr at room temperature, followed by two washes in TBS/tween buffer (5 min/each), secondary antibody (Novocastra Streptavidin-HRP, Leica Microsystems, Milan, Italy) for 30 min at room temperature, two washes in TBS/Tween (5 min/each) and then visualized using a 3,3′-diaminobenzidine. The primary antibody against PATZ1 is a previously described [17] rabbit polyclonal antibody raised against a peptide in the N-terminal region of the human PATZ1 protein (aa 1-276), diluted 1:500. The following negative controls were performed: (a) omission of the primary antibody; (b) substitution of the primary anti-serum with non-immune serum diluted 1:500 in blocking buffer; no immunostaining was observed after any in both cases. Sections were counterstained with hematoxylin and mounted. Stained tissue-microarray sections were evaluated by two different pathologists (RF, ADC) using uniform criteria. Discrepancies were resolved through simultaneous inspection and discussion of the results. Single-marker expression was recorded as low/high, after consideration of the expression in reactive compared with tumor cells and the specific cutoff of each marker (Table 4). The median immunopositivity was used as cutoff, corresponding to previous reported data [40–42], as reported in Table 4. The following optimal cutoff scores, based on the Youden index [41], were used for IHC staining associated with the public dataset, as previously reported [43]: 35% for CD10, 33% for BCL6, 45% for GCET1, 75% for FOXP1 and 58% for MUM1.

### RNA extraction and quantitative real time RT-PCR (RT-qPCR)

Total RNA was extracted from paraffin-embedded mouse tissues using the RNeasy FFPE Kit (Qiagen, Hilden, Germany), according to manufacturer's instructions. Reverse transcription was performed using random exanucleotides as primers and AMV reverse transcriptase (Life Technologies Italia, Monza, Italy) according to standard procedures. q-PCR analysis was carried out using the Power SYBR Green PCR Master Mix (Life Technologies Italia), according to manufacturer's instructions. Primers specific for the ribosomal protein S9 (*Rps9*) gene were used for normalization of the data. Primer sequences were as follows: *Patz1* (5′-GAGCTTCCCCGAGCTCAT-3′ / 5′-CAGATCTCGATGACCGACCT-3′); *Bcl6* (5′-GAAC TGTATGCAGATTCCAGTCA-3′ / 5′-ACTGTCCTCTTG TAATCCTTCCA-3′); *Bax* (5′-GTGAGCGGCTGCTTGTC T-3′ / 5′-GGTCCCGAAGTAGGAGAGGA-3′); *Rps9* (5′-C TGGACGAGGGCAAGATGAAGC-3′ / 5′-TGACGTTGG CGGATGAGCACA-3′).

### Statistical analysis and Kaplan-Meier survival curves

The Mann-Whitney test and the Pearson's *χ*^2^ test were used, where appropriate, to establish whether there were any relationships between the frequencies of PATZ1 expression in nuclear compartment and BCL6, BAX, histotypes and specific subtypes in DLBCL category.

Kaplan-Meier survival curves were used to analyze OS and PFS. In Figure 7, the stratification of High/Low PATZ1 was based on RNA expression using a cutoff of 73.3 according to the scan function of the R2 platform used (http://r2.amc.nl), where an optimum survival cutoff is established based on statistical testing (logrank test). In Figure 8, the stratification of High/Low PATZ1 was based on the median percentage of cells expressing nuclear PATZ1 protein by immunohistochemistry. Statistical significance was assessed by the logrank test. Multivariate analysis, through the Cox regression model, was performed to explore the influence of PATZ1 expression, cell of origin and IPI score on survival. A *P* value < 0.05 was regarded as significant for all analyses.

## SUPPLEMENTARY MATERIALS TABLE


