# Supplementary material for: PATZ1 expression correlates positively with BAX and negatively with BCL6 and survival in human diffuse large B cell lymphomas
Source: Oncotarget. 2016 Aug 1;7(37):59158–72. doi: 10.18632/oncotarget.10993 (PMC5312302; doi:10.18632/oncotarget.10993)
Supplement: Supplementary file 1 [file oncotarget-07-59158-s001.pdf]

PATZ1 expression correlates positively with BAX and negatively with BCL6 and survival in human diffuse large B cell lymphomas

Supplementary Materials

Supplementary Digital Content S1: Clinicopathological features and follow-up (f/u) in low PATZ1 DLBCL patients

patient ID[a]	gender	age	sub-type	BCL6	CD10	GCET1	FOXP1	MUM1	IPI code	IPI score	f/u
gsm776134	M	34	GCB	60	0	5	0	0	Low, int-low	1	2 months
gsm775986	M	68	ABC	100	0	0	100	100	high, int-high	3	2 months
gsm776472	M	59	ABC	5	0	5	80	70	high, int-high	4	2 months
gsm776132	M	61	ABC	60	0	10	10	5	low, int-low	2	6 months
gsm776276	F	68	ABC	80	0	5	90	80	high, int-high	3	6 months
gsm776181	F	53	ABC	5	0	0	70	60	high, int-high	4	7 months
gsm776026	F	74	GCB	100	100	35	50	10	low, int-low	2	10 months
gsm776062	M	80	ABC	80	20	90	90	90	low, int-low	2	11 months
gsm776218	M	60	ABC	80	15	20	100	40	high, int-high	4	11 months
gsm776243	M	76	GCB	75	70	60	5	80	high, int-high	4	11 months
gsm776250	M	73	GCB	80	60	30	90	75	high, int-high	3	11 months
gsm776072	M	53	GCB	90	100	0	100	30	low, int-low	1	15 months
gsm776393	M	18	GCB	75	0	90	0	0	low, int-low	2	16 months
gsm776320	M	84	GCB	70	75	80	0	10	low, int-low	1	21 months
gsm776077	M	74	ABC	100	5	90	100	100	high, int-high	4	23 months
gsm776354	F	54	GCB	80	89	10	25	0	unknown	uk	34 months
gsm776252	F	73	ABC	30	0	0	85	95	low, int-low	2	41 months
gsm776186	M	62	ABC	90	0	0	100	100	high, int-high	3	44 months
gsm776038	M	75	GCB	40	0	90	5	30	low, int-low	2	60 months

[a]from the GSE31312 dataset through the genomics analysis and visualization platform (http://r2.amc.nl).
